# Idiopathic Brainstem Neuronal Chromatolysis (IBNC): a novel prion protein related disorder of cattle?

**DOI:** 10.1186/1746-6148-4-38

**Published:** 2008-09-30

**Authors:** Martin Jeffrey, Belinda Baquero Perez, Stuart Martin, Linda Terry, Lorenzo González

**Affiliations:** 1 Veterinary Laboratories Agency (VLA-Lasswade), Pentlands Science Park, Bush Loan, Midlothian EH26 0PZ, UK; 2 VLA-Weybridge, Addlestone, Surrey, KT15 3NB, UK

## Abstract

**Background:**

The epidemic form of Bovine Spongiform Encephalopathy (BSE) is generally considered to have been caused by a single prion strain but at least two strain variants of cattle prion disorders have recently been recognized. An additional neurodegenerative condition, idiopathic brainstem neuronal chromatolysis and hippocampal sclerosis (IBNC), a rare neurological disease of adult cattle, was also recognised in a sub-set of cattle submitted under the BSE Orders in which lesions of BSE were absent. Between the years of 1988 and 1991 IBNC occurred in Scotland with an incidence of 7 cases per 100,000 beef suckler cows over the age of 6 years.

**Results:**

When the brains of 15 IBNC cases were each tested by immunohistochemistry, all showed abnormal labelling for prion protein (PrP). Immunohistological labelling for PrP was also present in the retina of a single case available for examination. The pattern of PrP labelling in brain is distinct from that seen in other ruminant prion diseases and is absent from brains with other inflammatory conditions and from normal control brains. Brains of IBNC cattle do not reveal abnormal PrP isoforms when tested by the commercial BioRad or Idexx test kits and do not reveal PrP^res ^when tested by Western blotting using stringent proteinase digestion methods. However, some weakly protease resistant isoforms of PrP may be detected when tissues are examined using mild proteinase digestion techniques.

**Conclusion:**

The study shows that a distinctive neurological disorder of cattle, which has some clinical similarities to BSE, is associated with abnormal PrP labelling in brain but the pathology and biochemistry of IBNC are distinct from BSE. The study is important either because it raises the possibility of a significant increase in the scope of prion disease or because it demonstrates that widespread and consistent PrP alterations may not be confined to prion diseases. Further studies, including transmission experiments, are needed to establish whether IBNC is a condition in which prion protein is abnormally regulated or it is yet a further example of an infectious cattle prion disease.

## Background

The transmissible spongiform encephalopathies, or prion diseases, are fatal neurodegenerative diseases characterized by the accumulation of a post-translationally modified variant of the host coded Prion protein (PrP). Until recently, only one form of naturally occurring cattle prion disease was recognized. However, extensive testing of sheep and cattle destined for the human food chain have recently revealed the presence of hitherto unsuspected variant forms of transmissible spongiform encephalopathy of cattle [1,2] and also of sheep [3].

Idiopathic brainstem neuronal chromatolysis and hippocampal sclerosis (IBNC) is a disorder of adult cattle which has some clinical similarity to bovine spongiform encephalopathy [4,5]. It was initially recognised from histological examination of cattle brains submitted as part of the UK statutory reporting of BSE suspects [6]. The disease is rare. In the period from 1988 to1991 it occurred at a rate of 7 cases per 100,000 beef suckler cows over the age of 6 years and 2.68 cases per 100,000 dairy cows of the same age [5]. The mean age of onset is 9 years with a range of 4 – 16 years. Most cases have been reported in Scotland and cases have also been diagnosed in England and Wales, but not from outside the UK. Most cases of IBNC occur singly on farms, but two farms have been identified which have experienced two cases each (MJ personal observations). The proportion of IBNC cases detected through the early 1990s was relatively consistent at 12–14% per year of the BSE negative case subset. During the peak of the BSE epidemic, 27 IBNC cases were recognized in Scotland in one year (MJ personal observations). IBNC cases continued to be found in recent years but there has been a fall in absolute numbers within the BSE negative subset. At least some IBNC cases have distinguishing clinical features from BSE [7] and the fall in histological diagnosis of IBNC cases may be a reflection of an increasingly critical appraisal of clinical signs when suspect BSE cases are examined in the field.

The pathological lesions of IBNC are distinctive and characterised by four types of histological change [4]. Neuronal degeneration and axonal degeneration involving brainstem and cranial nerve nuclei and radices of cranial nerves, accompanied by a non-suppurative inflammation proportionate to the degenerative changes, are invariably present. In approximately half the cases examined there is a spongiform change involving grey matter of medial and lateral geniculate nuclei, thalamus, hippocampus, striatum and cerebral cortex, together with hippocampal degeneration and sclerosis involving extensive loss of neurons [4]. The spongiform changes of IBNC involve neuroanatomical areas different from those vacuolated in BSE affected brains.

Testing for a number of different metabolic disorders, including vitamin B, vitamin E and selenium deficiency, the presence of antigens to Louping ill virus, Aujeszky's disease virus, Borna virus and Bovine Virus Diarrhoea virus failed to show any significant abnormalities (MJ personal observations). Immunohistochemical studies for PrP were initially performed in the mid-1990s on IBNC brain tissues using antibodies raised to murine PrP. No abnormal PrP was detected. Five brains were tested for scrapie associated fibrils by negative stain electron microscopy and were negative (unpublished data).

PrP immunohistochemistry tests on brains from IBNC cases performed during the 1990s were done using antibodies of low affinity for bovine PrP and methodologies that are of lower sensitivity than those currently available. Recent re-examination of tissues from IBNC cases using more sensitive labelling methods and antibodies capable of detecting lower levels of bovine PrP, consistently revealed PrP labelling in all cases tested. This present report describes the results of immunohistochemical and biochemical methods for PrP detection in a series of IBNC cases.

## Methods

Sixteen cases of IBNC were retrieved from the pathology archives at the VLA Lasswade laboratory. Cases were from cattle that were between 5 and 15 years of age when killed between 1993 and 2005. To control for inflammatory and degenerative changes and time of tissue preservation in paraffin wax, cases of malignant catarrhal fever, (a herpes virus infection of cattle), encephalic listeriosis, non-suppurative encephalitis, BSE and also, cattle brains with no significant morphological changes were also retrieved from the same archive. These latter cases had also been preserved in paraffin wax since 1992–1994.

Tissues of IBNC cases available for immunohistochemistry and, or, biochemistry are listed in table 1. For histology and immunohistochemical testing, whole brains were available from 9 IBNC cases: representative samples of medulla at the obex and cerebellar peduncles, midbrain, thalamus, striatum, cerebellum hippocampus and cereberal cortices were examined. From a single cow, additional tissues of eye, spleen, adrenal gland and lymph node were also examined. From the remaining seven cases only brainstem was available for testing.

**Table 1:** List of frozen and fixed IBNC tissues available for testing

*Animal Id*	*Fixed tissue*	*Frozen tissue*
2786/93	whole brain	Not available
3431/93	whole brain	Not available
3990/94	whole brain	Not available
3987/94	whole brain	Not available
3335/94	whole brain	Not available
3382/94	whole brain	Not available
0486/96	whole brain	Not available
2691/02	obex only	medulla only
1165/03	obex only	whole brain
2522/03	obex only	whole brain
2711/03	obex only	whole brain
2850/03	obex only	whole brain
2994/03	obex only	medulla only
5208/04	half brain	half brain
5193/04	obex only	whole brain
0522/05	whole brain	medulla only

The protocol for sampling suspect BSE cases has altered in the UK over time. Consequently, most IBNC cases from the early part of the epidemic lacked samples of frozen tissue for biochemical analyses. From the later part of the epidemic, only medulla was available for biochemical testing: from 2003 whole brains have been routinely frozen and retained. Samples of frozen brain were available from 7 cases on which a diagnosis of IBNC was established, based on the histology of medulla. From only one IBNC brain was half a brain available for biochemistry and half a brain available for histology. The tissues available for examination are listed in Table 1.

### Histology and immunohistochemistry

All brain sections available were stained with haematoxylin and eosin. Additional blocks of tissue of medulla, midbrain and thalamus were impregnated with silver according to Glees and Marsland's modification of Davenport's method for degenerate axons.

For immunohistochemistry, paraffin wax embedded tissues were sectioned at 5 μm, mounted on treated glass slides (Superfrost Plus; Menzel-Glaser, City, Germany) and dried overnight at 37°C. Initially, immunohistochemistry carried out in 1993–5 on IBNC cases used the 1B3 antibody, a polyclonal antibody which had been raised in rabbits to scrapie associated fibrils extracted from ME7 infected mouse brain. Methods used for epitope demasking employed only a formic-acid retrieval stage and did not use autoclaving. Subsequently, in 2007, immunohistochemistry was carried out as described by González et al [8]. Briefly, antigen retrieval included immersion of tissue sections in 98% formic acid for 5 min and autoclaving in 0.2% citrate buffer for 5 min at 121°C. After two blocking steps (to quench endogenous peroxidase activity and to remove non-specific tissue antigens), incubation with the primary antibody was carried out overnight at 4°C. Subsequent steps were performed using a commercial immunoperoxidase technique (Vector-elite ABC kit; Vector Laboratories, Peterborough, UK), after which sections were immersed in 0.5% copper sulphate, to enhance immunoperoxidase colour reaction. Finally, sections were counterstained with Mayer's haematoxylin. Seven PrP antibodies were used, all of which were first titrated on BSE infected sheep or cattle brains to determine the effective dilution range. The antibodies, their binding or eliciting sequences, and dilutions used are shown in table 2. All of these antibodies are considerably more sensitive for detecting bovine PrP than were antibodies used for IBNC labelling in 1993–5. Biotinylated antibodies were used for secondary enhancement: goat anti-rat was used to detect antibody R145 and a universal horse anti-mouse/rabbit was used to detect all other antibodies. For antibody controls, omission of the primary antibody and anti-isotype antibodies were also employed.

**Table 2:** Antibodies and dilutions used for immunohistochemistry and for biochemistry and their eliciting or mapped sequences.

*Antibody*	*sequence*	*reference*	*IHC*	*biochemistry*
12B2	^101^WGQGG^105#^	[19]	1:32000	NA
P4^¥^	^101^WGQGGSH^107#^	[20]	NA	0.4 μg/ml
12F10	^153^GSD....PNQ^-171^*	[21]	1:20000	NA
L42	^152^FGND......VYY^174 ^*	[22]	1:1000	NA
6H4	^155^DYEDRYYRE^163#^	[23]	1:800	NA
SHA31	^156^YEDRYYRE^163#^	[24]	NA	1:10
SAF84	^171^QVYYRPVDQY^181^*	[25]	1:2000	0.8 μg/ml
F99	^228^QYQRES^233#^	[26]	1:6000	2 μg/ml
R145	^231^RESQA^235#^	[19]	1:2000	NA

Codon numbers are according to the bovine PrP sequence where * indicates the eliciting sequence or ^# ^the mapped sequence.

¥ Antibody raised to sheep sequence which differs at one codon from the bovine. NA: not applied.

### Biochemical methods

#### TeSeE Western blotting

Sample extraction was carried out according to the manufacturer's instructions (Bio-Rad (California, USA) TeSeE Western Blot) with several modifications. In brief, brain tissue was ribolysed to give a 20% (w/v) homogenate. The homogenate was then incubated with DNAase (1/10 dilution at concentration of 2.5 mg/ml in 0.19 M MgCl_2_) at room temperature for 5 minutes. The samples were then digested with 0.3 units/ml proteinase K (Sigma-Aldrich, (Dorset, UK)); 0.3 units/ml is an in-house nomenclature and equivalent to 0.3 μl of Bio-Rad test proteinase K in terms of activity when compared with the TAME test (Pierce) or 4 μl/ml proteinase K (Bio-Rad) for 10 minutes, with digestion stopped by adding 1/25 Pefabloc SC (Fluka-Sigma-Aldrich, Dorset, UK) (46.7 mM in distilled H_2_O). Following precipitation and centrifugation at 15,000 g for 7 minutes, the pellets were incubated at 100°C for 5 minutes in 100 μl Laemmli solution (with 5% (v/v) beta-mercaptoethanol and 2% (w/v) SDS). A second centrifugation was performed at 15,000 g for 15 min. The supernatants were stored frozen at -20°C overnight. For analysis, the supernatants were heated at 100°C for 5 minutes, loaded on a 12% Criterion XT Bis-tris SDS gel (Bio Rad) and subjected to electrophoresis in NuPAGE running buffer (Invitrogen-California USA) at 200 V for 35 minutes. Proteins were transferred to a PVDF membrane (Bio-Rad) at 115 V for 60 min using NuPAGE transfer buffer.

Blots to be exposed to the SHA31 antibody were blocked for one hour with the solution provided by the manufacturers. Where antibodies F99, SAF84 and P4 were used, a blocking buffer of 5% milk powder in PBS supplemented with Tween 20 (PBST) was used. The membranes were incubated for one hour with the primary antibody: either SHA31 (Bio-Rad) 1/10 dilution in PBST, SAF84 (Spi Bio, Paris France) 0.8 μg/ml, P4 (R-BioPharm, Darmstadt, Germany) 0.4 μg/ml, or F99 2 μg/ml (VMRD, Inc. Pullman, Washington state, USA). The membranes were incubated with goat anti-mouse IgG antibody (Bio-Rad) conjugated to horseradish peroxidase diluted 1/10 in PBST. The membranes were visualized by chemiluminescence (ECL; Amersham, UK).

For the 4 μl/ml proteinase K concentration the addition of DNAase and pefabloc was omitted.

#### ELISA for determination of PrP^res^

20% homogenates were prepared as for the Western blot using the Bio-Rad protocol with the modifications described. The pellets obtained were solubilised by incubating at 100°C for 5 minutes in Reagent C. The method for the ELISA was carried out as described by the manufacturers. The absorbance was measured at 450 nm and 620 nm.

#### EIA for determination of aggregated PrP using a ligand based diagnostic test

For the determination of aggregated PrP in the absence of proteinase K the Idexx (Maine, USA) HerdChek BSE test was performed on samples according to the manufacturer's instructions with no modifications or deviations. Briefly samples were mixed with the working plate diluents and then loaded on a BSE antigen-capture EIA plate and incubated for 2.5 hours at room temperature. Aggregated PrP was observed using the conjugated anti-PrP antibodies provided with the kit. Absorbance was read at 450 nm and 620 nm.

#### Determination of positive values

The cut-off values for the Bio-Rad TeSeE ELISA and Idexx assays were calculated using the mean of the absorbance values of 90 confirmed BSE negative brainstem samples+ 3 standard deviations. This value was calculated as 0.166 Absorbance units (AU) for the TeSeE ELISA and 0.137 AU for the Idexx assay. Mean absorbance value and standard deviation using 0.3 μ/ml proteinase K for the Bio-Rad assay was 0.066 ± 0.033 (n = 90; range 0.022–0.198) and for the HerdChek assay 0.056 ± 0.027 (n = 90).

## Results

**Figure 1:**
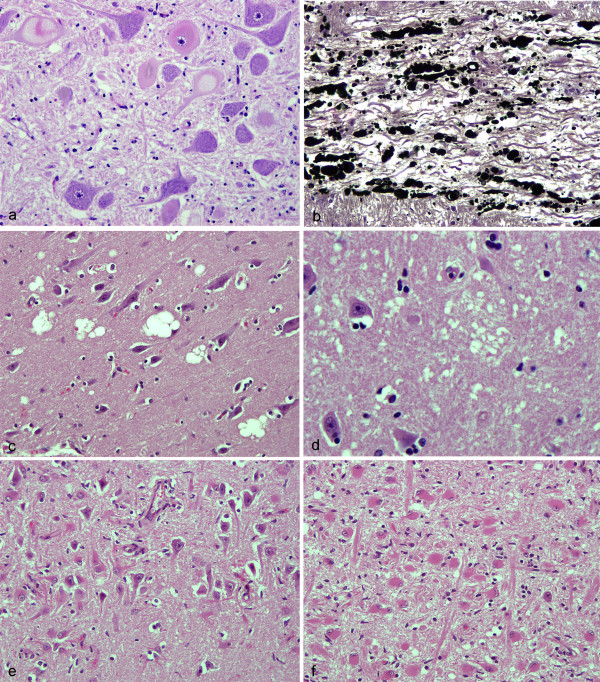
**Histopathology of IBNC**. 1a Case 522/05. Chromatolytic and degenerative changes of neurons of the DMNV HE mag ×225. 1b. Case 3382/94. Silver staining (black) showing extensive degeneration of myelinated axons in descending fibres of the radix of the facial nerve. Glees and Marsland mag ×250. 1c Case 3335/94. Loculated and foamy vacuolation of neuropil adjacent to and impinging on pyramidal neurons of the occipital cortex. HE mag ×250. 1d Case 522/05 Fine, lacy, vacuolation of neuropil in striatum. HE mag ×500. 1e Case 3382/94 Loss of neurons and reactive gliosis in the pyramidal neurons of the CA1 sector of the hippocampus HE mag ×210. 1f Case 3382/94 Gemistocytic replacement gliosis in the CA4 sector (dentate gyrus) of the hippocampus. HE mag ×240

The histology of each IBNC case was reviewed and the lesions seen were as previously described (Figure 1). Severe brainstem neuronal chromatolysis (Figure 1a), often accompanied by nuclear degeneration and occasional amphophilic, intranuclear inclusions, was present in several brainstem nuclei, being consistently present in the red nucleus, vestibular complex, dorsal motor nucleus of the vagal nerve and raphe. In addition, there was severe axonal and myelin degeneration, prominently affecting the radices and roots of cranial nerves (Figure 1b). The degenerative changes of neurons were accompanied by marked gliosis of parallel severity, and proceeding to gemistocytosis and non-suppurative inflammation of meninges and perivascular spaces. Marked spongiform change with neuronal degeneration was present in the midbrain, thalamus, striatum and cortex. Not fully described in previous papers are the different patterns of vacuolation found in IBNC. Three patterns of vacuolation were recognized: firstly a large loculated (foamy) vacuole typically found in the Betz cell layer of the cerebral cortex (Figure 1c) and also in the thalamus. Single or multiple, round or ovoid, grey matter vacuoles of a form and character similar to that of scrapie or BSE were present in the midbrain, thalamus and striatum. These lesions were not present in medulla and were specifically absent from BSE target sites. Thalamic vacuoles were sometimes focally very numerous and sometimes associated with neuronal degeneration. These two forms of vacuolation were present in 6 of 9 cases where whole brain was available for examination. A third vacuolar change, in which the neuropil was pale and showed a diffuse lacy appearance or very small vacuoles was also found (Figure 1d). Sub-total loss of CA1 and CA2 pyramidal neurons and neurons of the dentate gyrus, was present in 3 of the 9 brains where hippocampus samples were available for examination (Figure 1e, f). No significant morphological lesions were recognised in eye or viscera.

The effective operating dilution for each of the antibodies was determined for BSE affected sheep or cattle brains and is listed in Table 2. The efficiency of each antibody for detection of disease specific PrP found in cattle BSE differed with F99, 6C2, SAF84 and 12F10 all producing high intensity labelling. PrP labelling of sections from IBNC cases was obtained with all antibodies but the ranking of sensitivity of detection of PrP in IBNC cases and disease specific PrP in cattle BSE cases was not the same. In particular 6C2, which give strong labelling of disease specific PrP accumulations in BSE affected cattle, gave weak labelling in IBNC cases. F99 and SAF84 labeled some 'dark' neurons in control brains tissues and F99 produces some weak, diffuse neuropil labelling. All other antibodies were 'clean' with no background staining. IBNC cases showed a distinctive pattern of PrP labelling that was present in sections of all IBNC cases but was absent from brains of all infectious or inflammatory conditions, brains with no significant lesions and brains from BSE cases. The same staining patterns were found in material which had been preserved in wax or in fixative for widely differing time intervals; it was reproduced in replicate staining runs and identical patterns were found with different antibodies. Thus, we consider that this labelling represents an abnormal form of PrP labelling. However, immunohistochemical methods alone do not reveal whether such accumulations are due to increased expression or altered distribution of normal forms of the protein, or whether they are PrP accumulations that are abnormal in conformation or aggregation, as they are in BSE and other prion diseases.

PrP labelling was detected in all IBNC cases though not in every case at all sites examined (Table 3). F99, SAF84 and L42 antibodies gave the greatest amounts of labelling. In most cases labelling was widespread throughout the brain. In three cases labelling was more or less confined to the striatum and in one of these three, the labelling was restricted to the putamen. HE stained sections of these three cases did not show any large loculated neuropil vacuoles. All other cases gave a wide distribution of labelling including medulla, cerebellum, midbrain, thalamus, striatum, hippocampus and cerebrum. In brainstem sections, labelling was present in the spinal tract nucleus of the trigeminal nerve and in the cerebellum, most labelling involved the cerebellar molecular layer.

**Table 3:** showing animals tested, age, tissues available for biochemical testing and the presence and absence of PrP by immunohistochemistry.

*Animal Id*	*Age*	*immunohistochemistry*		*Routine*	*Immunoblot*	
		medulla	whole brain	BioRad	medulla	whole brain
2786/93	7	pos	pos	NA	NA	NA
3431/93	8	negative	pos	NA	NA	NA
3990/94	5	pos	pos	NA	NA	NA
3987/94	10	negative	pos	NA	NA	NA
3335/94	8	pos	pos	NA	NA	NA
3382/94	10	pos	pos	NA	NA	NA
0486/96	NA	pos	pos	NA	NA	NA
2691/02	15	negative	NA	Neg	yes	NA
1165/03	12	pos	NA	Neg	yes	yes
2522/03	13	pos	NA	Neg	yes	yes
2711/03	10	pos	NA	Neg	yes	yes
2850/03	8	pos	NA	Neg	yes	yes
2994/03	12	pos	NA	Neg	yes	NA
5208/04	12	negative	pos	Neg	yes	yes
5193/04	13	pos	NA	Neg	yes	yes
0522/05	13	pos	pos	Neg	NA	NA

NA tissue or data not available

**Figure 2:**
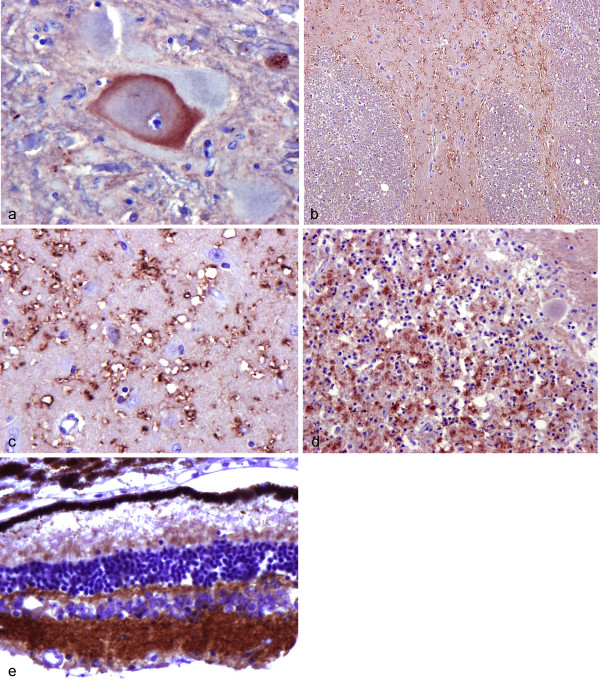
**PrP immunohistochemistry in IBNC**. 2a Case 1165/03 A degenerate neuron in the DMNV shows intracytoplasmic labelling for PrP. F99 antibody mag ×550. 2b Case 522/05. PrP labelling is present in grey matter of the striatum. The white matter is unlabelled. F99 antibody mag ×40. 2c Case 3431/93 Detail of grey matter labelling of the caudate nucleus showing the association of labelling with the rims of vacuoles and of the neuropil adjacent to small vacuoles. F99 antibody mag ×480. 2d Case 2786/93 Diffuse labelling of the glomerular zones of neuropil in the cerebellar granule cell layer. F99 antibody mag ×200. 2e Case 486/96 Retina showing PrP labelling of the inner and outer plexiform layers of the sensory retina. F99 antibody mag ×120.

Most PrP labelling was found in grey matter (Figure 2b) in the form of globular, ring or in 'C' shaped patterns (Figure 2c). Occasionally, several of these were arranged in a line. Very often this pattern of labelling was present at the rim of small vacuoles (Figure 2c). Some bundles of white matter in which there was strong vacuolation at the grey matter interface were also strongly PrP labelled at this interface with the above pattern. The intensity of PrP labelling was greatest in those cases in which lesions of degeneration and spongiform change were most marked. When the sites of labelling were compared with HE stained sections this pattern of labelling corresponded to the 'lacy' neuropil type of micro-vacuolation, both in specific location within individual sections and in overall brain distribution. The larger loculated or foamy forms of vacuoles and more typical scrapie like vacuoles were not specifically labelled.

In the cerebellum of two cases diffuse labelling was found in the granular layer and neuropil between granule cell neuronal nuclei (Figure 2d). Diffuse labelling of both inner and outer plexiform layers was found in the one eye (Figure 2e) examined but no labelling was found in the limited range of viscera. Rarely, degenerate chromatolytic neurons showed intracytoplasmic labelling (Figure 2a).

### Biochemistry

From 2002 all cattle taken under the BSE Regulations were routinely tested by the BioRad ELISA. The records of 7 IBNC cases from within this period were located. All results were recorded as negative. Repeat testing of samples using standard commercial BioRad and Idexx test kits also gave negative results.

The results of BioRad tests using reduced levels of proteinase K are shown in table 4. Of the total of 24 IBNC samples of different brain sites tested, 15 (62.5%) gave values above those of the test kit negative control and also above the BSE negative brain pool control. Seven of these samples (29%) were above the calculated cut off value. Values above and up to 2 times greater than the calculated cut off were found for each case but not for each brain site. Individual samples from two brains (2691/02 and 1165/03) which did not initially show results above the calculated cut off values did so on re-testing (data not shown).

**Table 4:** BioRad Elisa tissues results under conditions of mild protease digestion

Sample ID	tissue site	0.3 [PK] ELISA
2691/02	brain stem	0.088
1165/03	cortex	0.122
1165/03	cerebellum	0.055
1165/03	midbrain	0.078
1165/03	brain stem	0.151
2522/03	cortex	0.083
2522/03	cerebellum	**0.285**
2522/03	midbrain	**0.334**
2711/03	cortex	**0.210**
2711/03	cerebellum	**0.241**
2711/03	midbrain	0.153
2711/03	brain stem	0.101
2850/03	cortex	0.136
2850/03	cerebellum	0.032
2850/03	midbrain	**0.266**
2850/03	brain stem	0.146
5193/04	cortex	0.101
5193/04	cerebellum	0.041
5193/04	midbrain	**0.229**
5193/04	brain stem	0.063
5208/04	cortex	0.094
5208/04	cerebellum	0.049
5208/04	midbrain	0.122
5208/04	brain stem	**0.194**

-ve BSE brain pool control		0.100
-ve kit control		0.011
Calculated cut off value		0.165

Values in bold are above the calculated cut off.

**Figure 3:**
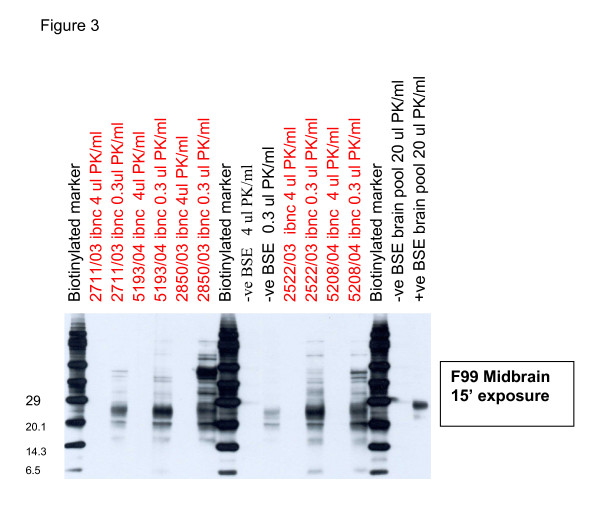
**PrP immunoblots on IBNC and BSE cases**. Western blot of 5 different IBNC midbrain samples digested with 4 μl (lanes 2,4,6,11, 13) or 0.3 μl/ml (lanes 3,5,7,12,14) of proteinase K. An individual control brain digested with 4 μ (lane 9,) or 0.3 μl/ml (lane10) proteinase K and a control brain (lane 16) and pooled BSE brains (lanes 17) digested with 20 μl/ml of proteinase K are also present. Molecular markers are at lanes 1,8, and 15. In each case 4 μl/ml proteinase K results in complete digestion of PrP. Although residual PrP^c ^is present in control brain each of the IBNC brains gives a stronger signal and multiple labelled bands when developed with the F99 antibody at 15 minutes exposure.

Western blots were carried out on the samples shown in table 3. In each case no residual protease resistant PrP (PrP^res^) was found when 20 or 4 μl/ml of proteinase K was used. When 0.12 μl/ml or 0.3 μl/ml of proteinase K was used, a signal was detected on the blots of all samples including those of the negative controls. IBNC samples were indistinguishable from negative controls with digestions of 0.12 μl/ml proteinase K. However, when 0.3 μl/ml of proteinase K was used more residual PrP was detected in IBNC cases than in the controls (Figure 3) and with each of the antibodies tested (only illustrations of F99 are shown).

## Discussion

This study shows that the novel condition of cattle previously identified as IBNC and recognized from within the BSE suspect submissions, abnormally expresses or accumulates PrP in brain and retina. However, this abnormal PrP is not composed of isoforms that are strongly resistant to protease digestion suggesting that it is not present in the form of large aggregates.

Immunohistochemical demonstration of PrP labelling and increased levels of PrP mRNA have previously been described in adult humans affected with acute vascular disorders, in infants with perinatal hypoxia and experimental infarction of rodents [9]. These findings are considered to represent upregulation of PrP expression which encompasses part of the oxidative stress response of neurons. We have also observed increased PrP in the cytoplasm of neurons undergoing ischaemic degeneration in a variety of sheep encephalopathies. Though ischaemic neuronal degeneration is not a feature of IBNC, nevertheless, the presence of PrP within the cytoplasm of some chromatolytic and degenerate neurons of IBNC affected cattle is consistent with the idea that stressed neurons may respond by increasing PrP expression.

Though present in only two cows, the pattern of PrP accumulation within the granule cell layer of the cerebellum is morphologically similar to that reported by several authors for Nor 98 types of the transmissible spongiform encephalopathy or prion disease of sheep [3,10]. The PrP accumulation within the plexiform layers of the eye is similar to that of both natural scrapie [11] and of Nor 98 (MJ personal observations). However the majority type of PrP labelling that occurred in all IBNC cases was found within the neuropil, mainly in the rostral neuraxis and cerebrum, the nature of which is previously unreported in cattle or in any other prion disorder. This novel pattern of labelling appears to correspond to a rarefaction or fine microvacuolation of neuropil as seen on standard HE stained sections.

Pathological, biochemical and bioassay data all suggest that the epidemic form of cattle BSE is a single strain. However, recent large scale EU wide surveillance for BSE has led to the unexpected discovery of rare and hitherto unknown prion diseases of cattle. Small numbers of atypical forms of cattle prion diseases have now been recognized from several European countries, in the USA and Japan and can be distinguished by histological, molecular and transmission characteristics [12-14]. Bovine Amyloidotic Spongiform Encephalopathy (BASE) was the first of these novel cattle prion disorders to be recognized and was characterized by the presence of numerous small amyloid deposits of abnormal PrP. It was initially discovered in three aged Italian cattle [1] and has subsequently been transmitted to transgenic mice [13,14]. A further variant of a cattle prion disease affecting cows between 8 and 15 years was initially recognized in France [2] and has also been transmitted to mice. BSE and these novel cattle prion diseases can be distinguished using biochemical and molecular methods and are now classified as C, H and L type isolates [12]. H type and L type (BASE) isolates are defined according to the higher and lower positions of the unglycosylated PrP^res ^bands in Western blots, respectively, when compared to the position of the corresponding band in classical BSE (C type) isolates [12]. L type cases formerly classified as BASE, have a distinctive glycopattern in which monoglycoslyated PrP^res ^predominates compared to BSE [12,14]. While IBNC cases are on average older than BSE cases they occupy a similar age class of cattle to that of H and L type cattle prion diseases, but IBNC can be readily distinguished from H, L and C type cattle prion disease by morphologic pathology and by the absence of PrP^res ^under stringent conditions of protease digestion.

Not all abnormal PrP^res ^isoforms detected from brains of animals affected with prion disease are resistant to stringent protease digestion. The PrP^res ^of two sheep of the ARR/ARR PrP genotype affected with a classical scrapie-like disease accumulated unusually protease sensitive isoforms of PrP [15]. Similarly, the transmissible prion disease of sheep known as Nor 98 and related conditions (often referred to as atypical scrapie) also have weakly protease resistant PrP^res ^[3,16]. Nor 98 does not appear to transmit readily to other sheep under field conditions: it also does not transmit to conventional mice although it does readily transmit disease to one strain of transgenic mouse which substantially over-expresses the VRQ allele of sheep PrP [16,17]. The transgenic PG14 mouse also has PrP^res ^which is even more readily digested than that found in Nor 98 but this prion protein disorder has not so far been successfully transmitted [18]. The biochemical analyses of limited numbers of IBNC cases clearly shows that highly aggregated forms of protease resistant PrP are not present in brain tissue. However, when the data from the ELISA and immunoblot tests using mild protease digestion are compared with that of normal control material it is possible that smaller aggregates of PrP molecules may be present.

## Conclusion

The present results indicate that there are changes in PrP expression or accumulation in the neurodegenerative cattle disorder known as IBNC. The pathology and biochemistry of IBNC are quite distinct from that of other prion diseases of cattle and other species but the pathology does include grey matter spongiform changes. The transmissibility of this disorder is undetermined. These results are interesting as they show that either the range of prion diseases and associated pathology is still wider than previously thought or that substantial abnormalities of prion protein expression may be associated with brain lesions unconnected with classical prion diseases. Further biochemical and transmission studies are needed to determine which of these possibilities is correct.

## Authors' contributions

MJ, LG and SM, performed the histology and immunohistochemistry. BBP performed the biochemical analyses and both BBP and LT analyzed and interpreted the biochemical studies. MJ drafted the manuscript with contributions from all other authors.

## References

[B1] Casalone C, Zanusso G, Acutis P, Ferrari S, Capucci L, Tagliavini F, Monaco S, Caramelli M (2004). Identification of a second bovine amyloidotic spongiform encephalopathy: Molecular similarities with sporadic Creutzfeldt – Jakob disease. PNAS USA.

[B2] Biacabe AG, Laplanche JL, Ryder S, Baron T (2004). Distinct molecular phenotypes in bovine prion diseases. Embo Rep.

[B3] Benestad SL, Sarradin P, Thu B, Schonheit J, Tranulis MA, Bratberg B (2003). Cases of scrapie with unusual features in Norway and designation of a new type, Nor98. Vet Rec.

[B4] Jeffrey M (1992). A neuropathological survey of brains submitted under the bovine spongiform encephalopathy orders in Scotland. Vet Rec.

[B5] Jeffrey M, Wilesmith JW (1992). Idiopathic brainstem neuronal chromatolysis and hippocampal sclerosis: a novel encephalopathy in clinically suspect cases of bovine spongiform encephalopathy. Vet Rec.

[B6] Jeffrey M (1990). Neurohistopathological observations on Bovine spongiform encephalopathy submissions in Scotland. State Veterinary Journal.

[B7] Stewart M (1997). Idiopathic brainstem neuronal chromatolysis in cattle: two case studies. Vet Rec.

[B8] Gonzalez L, Martin S, BegaraMcGorum I, Hunter N, Houston F, Simmons M, Jeffrey M (2002). Effects of agent strain and host genotype on PrP accumulation in the brain of sheep naturally and experimentally affected with scrapie. J Comp Pathol.

[B9] McLennan NF, Brennan PM, McNeill A, Davies I, Fotheringham A, Rennison KA, Ritchie D, Brannan F, Head MW, Ironside JW, Williams A, Bell JE (2004). Prion protein accumulation and neuroprotection in hypoxic brain damage. Amer J Pathol.

[B10] Buschmann A, Luhken G, Schultz J, Erhardt G, Groschup MH (2004). Neuronal accumulation of abnormal prion protein in sheep carrying a scrapie-resistant genotype (PrPARR/ARR). J Gen Virol.

[B11] Hortells P, Monzon M, Monleon E, Acin C, Vargas A, Bolea R, Lujan L, Badiola JJ (2006). Pathological findings in retina and visual pathways associated to natural scrapie in sheep. Brain Res.

[B12] Jacobs JG, Langeveld JP, Biacabe AG, Acutis PL, Polak MP, Gavier-Widen D, Buschmann A, Caramelli M, Casalone C, Mazza M, Groschup M, Erkens JH, Davidse A, Van Zijderveld FG, Baron T (2007). Molecular discrimination of atypical bovine spongiform encephalopathy strains from a geographical region spanning a wide area in Europe. J Clin Microbiol.

[B13] Capobianco R, Casalone C, Suardi S, Mangieri M, Miccolo C, Limido L, Catania M, Rossi G, Di Fede G, Giaccone G, Bruzzone MG, Minati L, Corona C, Acutis P, Gelmetti D, Lombardi G, Groschup MH, Buschmann A, Zanusso G, Monaco S, Caramelli M, Tagliavini F (2007). Conversion of the BASE prion strain into the BSE strain: the origin of BSE?. PLoS Pathog.

[B14] Buschmann A, Gretzschel A, Biacabe AG, Schiebel K, Corona C, Hoffmann C, Eiden M, Baron T, Casalone C, Groschup MH (2006). Atypical BSE in Germany – proof of transmissibility and biochemical characterization. Vet Microbiol.

[B15] Groschup MH, Lacroux C, Buschmann A, Luhken G, Mathey J, Eiden M, Lugan S, Hoffmann C, Espinosa JC, Baron T, Torres JM, Erhardt G, Andreoletti O (2007). Classic scrapie in sheep with the ARR/ARR prion genotype in Germany and France. Emerg Infect Dis.

[B16] Le Dur A, Beringue V, Andreoletti O, Reine F, Lan Lai T, Baron T, Bratberg B, Vilotte JL, Sarradin P, Benestad SL, Laude H (2005). A novel type of prion naturally infects scrapie-resistant sheep. PNAS.

[B17] Simmons MM, Konold T, Simmons HA, Spencer YI, Lockey R, Spiropoulos J, Everitt S, Clifford D (2007). Experimental transmission of atypical scrapie to sheep. BMC Vet Res.

[B18] Chiesa R, Piccardo P, Quaglio E, Drisaldi B, SiHoe SL, Takao M, Ghetti B, Harris DA (2003). Molecular distinction between pathogenic and infectious properties of the prion protein. J Virol.

[B19] Jeffrey M, Gonzalez L, Chong A, Foster J, Goldmann W, Hunter N, Martin S (2006). Ovine infection with the agents of acrapie (CH1641 Isolate) and bovine spongiform encephalopathy: immunochemical similarities can be resolved by immunohistochemistry. J Comp Pathol.

[B20] Thuring CM, Erkens JH, Jacobs JG, Bossers A, van Keulen LJ, Garssen GJ, Van Zijderveld FG, Ryder SJ, Groschup MH, Sweeney T, Langeveld JP (2004). Discrimination between scrapie and bovine spongiform encephalopathy in sheep by molecular size, immunoreactivity, and glycoprofile of prion protein. J Clin Microbiol.

[B21] Krasemann S, Jurgens T, Bodemer W (1999). Generation of monoclonal antibodies against prion proteins with an unconventional nucleic acid-based immunization strategy. J Biotechnol.

[B22] Harmeyer S, Pfaff E, Groschup MH (1998). Synthetic peptide vaccines yield monoclonal antibodies to cellular and pathological prion proteins of ruminants. J Gen Virol.

[B23] Korth C, Stierli B, Streit P, Moser M, Schaller O, Fischer R, Schulzschaeffer W, Kretzschmar H, Raeber A, Braun U, Ehrensperger F, Hornemann S, Glockshuber R, Riek R, Billeter M, Wuthrich K, Oesch B (1997). Prion (PrPsc)-specific epitope defined by a monoclonal-antibody. Nature.

[B24] Feraudet C, Morel N, Simon S, Volland H, Frobert Y, Creminon C, Vilette D, Lehmann S, Grassi J (2005). Screening of 145 anti-PrP monoclonal antibodies for their capacity to inhibit PrPSc replication in infected cells. J Biol Chem.

[B25] Demart S, Fournier JG, Creminon C, Frobert Y, Lamoury F, Marce D, Lasmezas C, Dormont D, Grassi J, Deslys JP (1999). New insight into abnormal prion protein using monoclonal antibodies. Biochem Biophys ResCommun.

[B26] O'Rourke KI, Baszler TV, Besser TE, Miller JM, Cutlip RC, Wells GAH, Ryder SJ, Parish SM, Hamir AN, Cockett NE, Jenny A, Knowles DP (2000). Preclinical diagnosis of scrapie by immunohistochemistry of third eyelid lymphoid tissue. J Clin Microbiol.

